# Iron–Immune Crosstalk at the Maternal–Fetal Interface: Emerging Mechanisms in the Pathogenesis of Preeclampsia

**DOI:** 10.3390/antiox14070890

**Published:** 2025-07-19

**Authors:** Jieyan Zhong, Ruhe Jiang, Nan Liu, Qingqing Cai, Qi Cao, Yan Du, Hongbo Zhao

**Affiliations:** 1 Obstetrics & Gynecology Hospital of Fudan University, Shanghai 200433, China; 23211250024@m.fudan.edu.cn (J.Z.); 23211250010@m.fudan.edu.cn (R.J.); 24111250022@m.fudan.edu.cn (N.L.); qingqingcai@fudan.edu.cn (Q.C.); qicao@fudan.edu.cn (Q.C.); 2 Shanghai Key Lab of Reproduction and Development, Shanghai 200433, China; 3 Shanghai Key Lab of Female Reproductive Endocrine Related Diseases, Shanghai 200433, China

**Keywords:** ferroptosis, macrophage, preeclampsia, maternal–fetal interface, iron metabolism, iron–immune axis

## Abstract

Preeclampsia (PE) is a pregnancy-specific hypertensive disorder characterized by systemic inflammation, endothelial dysfunction, and placental insufficiency. While inadequate trophoblast invasion and impaired spiral artery remodeling have long been recognized as central to its pathogenesis, emerging evidence underscores the critical roles of dysregulated iron metabolism and its crosstalk with immune responses, particularly macrophage-mediated inflammation, in driving PE development. This review systematically explores the dynamic changes in iron metabolism during pregnancy, including increased maternal iron demand, placental iron transport mechanisms, and the molecular regulation of placental iron homeostasis. We further explore the contribution of ferroptosis, an iron-dependent form of regulated cell death driven by lipid peroxidation, to trophoblast dysfunction and pregnancy-related diseases, including PE. Macrophages, pivotal immune regulators at the maternal–fetal interface, exhibit distinct polarization states that shape tissue remodeling and immune tolerance. We outline their origin, distribution, and polarization in pregnancy, and emphasize their aberrant phenotype and function in PE. The bidirectional crosstalk between iron and macrophages is also dissected: iron shapes macrophage polarization and function, while macrophages reciprocally modulate iron homeostasis. Notably, excessive reactive oxygen species (ROS) and pro-inflammatory cytokines secreted by M1-polarized macrophages may exacerbate trophoblast ferroptosis, amplifying placental injury. Within the context of PE, we delineate how iron overload and macrophage dysfunction synergize to potentiate placental inflammation and oxidative stress. Key iron-responsive immune pathways, such as the HO-1/hepcidin axis and IL-6/TNF-α signaling, are discussed in relation to disease severity. Finally, we highlight promising therapeutic strategies targeting the iron–immune axis, encompassing three key modalities—iron chelation therapy, precision immunomodulation, and metabolic reprogramming interventions—which may offer novel avenues for PE prevention and treatment.

## 1. Introduction

Preeclampsia, as clinically defined by the American College of Obstetrics and Gynecology (ACOG), is primarily characterized by elevated blood pressure (systolic ≥ 140 mm Hg or diastolic ≥ 90 mm Hg on 2 occasions, 4 h apart in a previously normotensive woman) accompanied by proteinuria (≥300 mg/24-h urine collection, protein/creatinine ratio ≥0.3, or dipstick reading of 1+). It may also present with severe symptoms, including systolic blood pressure ≥ 160 mm Hg or diastolic ≥ 110 mm Hg on 2 occasions, 4 h apart while on bed rest; thrombocytopenia (<100,000/μL); liver function tests 2× the normal value or severe, persistent right upper quadrant or epigastric pain; serum creatinine concentration > 1.1 mg/dL or the doubling of creatinine in the absence of other renal disease; pulmonary edema; and new-onset cerebral or visual symptoms [1]. Preeclampsia is a serious obstetric complication, causing approximately 70,000 maternal deaths and 500,000 perinatal deaths worldwide each year. It significantly contributes to increased maternal and perinatal mortality, cesarean deliveries, preterm births, and admissions to intensive care units [2].

The pathogenesis of preeclampsia is classically described as a two-stage process initiated by placental abnormalities. Stage 1 involves shallow placental implantation and poor uterine spiral artery remodeling due to trophoblast dysfunction, triggered by genetic, environmental, and immunological factors, ultimately leading to placental ischemia. Mechanisms involved may include oxidative stress, placental hypoxia, abnormalities in heme oxygenase or other enzymes, and damage to the placenta by natural killer (NK) cells [3]. The second stage refers to the maternal systemic response, where placental ischemia triggers widespread endothelial dysfunction through multiple pathways: imbalances of circulating angiogenic factors, alterations in inflammatory cytokines and immune cells, and dysregulation of the renin-angiotensin system and sympathetic nervous system. Clinically, this stage is characterized by systemic vascular dysfunction, proteinuria or glomerular endotheliosis, hypertension, visual disturbances, headaches, cerebral edema or seizures (eclampsia), hemolysis, elevated liver enzymes, and low platelet count (HELLP) syndrome, and coagulation abnormalities [3].

In recent years, accumulating evidence has indicated that disturbances in iron metabolism and immune homeostasis may constitute critical pathogenic factors in preeclampsia (PE), extending beyond conventional mechanistic understandings. During normal pregnancy, maternal iron metabolism undergoes dynamic adaptations to meet the growing demands of the fetus. Iron is actively transported across the placenta to the fetal circulation while maintaining redox homeostasis within the maternal–fetal interface. However, multiple studies have reported systemic and placental iron overload in PE patients, evidenced by elevated serum ferritin concentrations and increased transferrin saturation, implying that disrupted iron homeostasis may play a significant role in the disease pathogenesis. Excessive iron accumulation potentiates oxidative stress, accelerates lipid peroxidation, and triggers cellular injury, thereby collectively exacerbating placental dysfunction. Simultaneously, immune dysregulation at the maternal–fetal interface has also been established as another fundamental element in PE pathogenesis. Among immune cells, macrophages not only represent the predominant immune cell population but also perform essential functions in sustaining immune tolerance, promoting spiral artery remodeling, and eliminating apoptotic cell debris. In PE, decidual macrophages frequently exhibit a polarization imbalance, characterized by an overabundance of pro-inflammatory M1 subsets and diminished anti-inflammatory M2 populations, thereby fostering a pro-inflammatory microenvironment that aggravates tissue injury. Notably, researchers have increasingly elucidated the sophisticated reciprocal interplay between iron metabolism and macrophage activity. Iron availability can directly influence macrophage polarization states and functional phenotypes, while macrophages conversely maintain local iron equilibrium by regulating the expression of iron transport and storage proteins, including transferrin receptors, ferritin, and ferroportin. This emerging iron–macrophage axis is now considered a plausible unifying mechanism that integrates metabolic dysregulation, immune imbalance, and PE progression.

## 2. Iron Metabolism in Pregnancy

### 2.1. Maternal Iron Demand and Systemic Adaptation

Iron plays a crucial role in various physiological processes, including erythropoiesis, oxygen transport, DNA synthesis, hormone production, energy metabolism, immune regulation, and neurodevelopment. For non-pregnant individuals, the daily iron requirement is typically around 20 mg, necessary to support these functions [4]. During pregnancy, the maternal iron demand increases by approximately 30%, principally to accommodate expanded blood volume, support maternal and fetal hematopoiesis, facilitate fetal development, and establish initial fetal iron stores [5,6]. According to the World Health Organization (WHO), anemia affects >40% of pregnant women worldwide, with iron deficiency accounting for >50% of these cases [7]. As the most prevalent nutritional deficiency worldwide, iron deficiency anemia demonstrates significant associations with pregnancy complications, including preterm birth (PTB), low birth weight (LBW), fetal growth restriction (FGR), and impaired fetal neurodevelopment and immune function [8,9]. Consequently, this evidence base has established iron supplementation as standard antenatal care. The physiological trajectory of pregnancy involves cessation of menstruation (reducing iron losses) coupled with progressive hepcidin suppression during the later stages of pregnancy. This endocrine adaptation facilitates tissue iron mobilization to satisfy the increased maternal serum iron requirements. These physiological adaptations may impair the body’s ability to regulate iron levels effectively [10]. Recent studies have raised concerns about routine iron supplementation during pregnancy, proposing that supranormal iron administration in replete women may be associated with an risk of obstetric complications, particularly preeclampsia [11].

### 2.2. Iron Transport Mechanisms Across the Placenta

At the maternal–fetal interface, iron is actively transported from the maternal circulation to the fetus via syncytiotrophoblasts (STBs), a process essential for supporting fetal growth and development [12]. While transferrin-bound iron (TBI) serves as the predominant iron source, alternative forms including non-transferrin-bound iron (NTBI), heme iron, and ferritin also contribute to placental iron acquisition [13]. The canonical transferrin-mediated pathway involves several sequential steps: (1) Fe^3+^-loaded transferrin-bound (holo-Tf) binds to transferrin receptor 1 (TfR1) expressed on the apical membrane of STBs, initiating clathrin-mediated endocytosis [14,15]. (2) The internalized iron-Tf complex undergoes vesicular trafficking to early endosomes where Fe^3+^ is reduced to Fe^2+^ by six-transmembrane epithelial antigen of the prostate 3 (STEAP3) and then transported across the endosomal membrane into the STB cytoplasm through divalent metal transporter 1 (DMT1, also known as SLC11A2). (3) Intracellular iron handling in STBs involves two principal fates: storage or export. For iron storage, Fe^2+^ is incorporated into ferritin nanocages (composed of ferritin heavy chain 1 (FTH1) and ferritin light chain (FTL) subunits) to sequester potentially harmful free iron and mitigate oxidative stress from ROS. Alternatively, for transplacental transfer, Fe^2+^ is exported across the basolateral membrane into the fetal stroma through ferroportin (FPN), the sole known mammalian iron exporter (SLC40A1). Following basolateral efflux, iron traverses the fetal endothelium to ultimately reach the fetal circulation [16,17].

However, the functional significance of DMT1 in intracellular iron transport remains controversial. While DMT1 is well-established as essential for intestinal absorption and erythropoiesis, its function may be non-essential in placental and hepatic systems, as SLC11A2 knockout mice display comparable hepatic iron storage levels to wild-type mice, suggesting the existence of compensatory mechanisms for maternal–fetal iron transfer independent of DMT1. Nevertheless, the severe anemia observed in these knockout models indicates DMT1 likely plays a non-redundant role in placental iron transport, warranting further investigation [18].

In addition to DMT1, the placenta expresses alternative iron transporters that become particularly important during iron overload conditions. Zrt- and Irt-like protein 8 (ZIP8, SLC39A8) and ZIP14 (SLC39A14) facilitate the uptake of NTBI, with distinct species-specific expression patterns: while ZIP14 is uniquely expressed in the mouse placenta, ZIP8 shows predominant expression in human placenta [19,20]. Slc39a14 knockout mice exhibit growth retardation, whereas Slc39a8 deficiency results in embryonically lethal, underscoring ZIP8’s essential function in early embryogenesis [21,22].

The final step of transplacental iron transfer requires oxidation of Fe^2+^ to Fe^3+^ to be absorbed by fetal vascular endothelial cells following FPN-mediated export from STBs. This critical conversion is catalyzed by multicopper ferroxidases in the placenta, including zyklopen (ZP/HEPHL1), ceruloplasmin (CP), and hephaestin (HEPH), which collectively maintain appropriate iron redox states for fetal uptake.

Within STBs, intracellular Fe^2+^ concentrations exhibit a critical association with ferroptotic pathways. Nuclear receptor coactivator 4 (NCOA4)-mediated ferritinophagy orchestrates the degradation of ferritin, liberating Fe^2+^, elevating the labile iron pool. Excess iron catalyzes the generation of ROS through the Fenton reaction, ultimately driving the ferroptotic cascade [17]. To counteract iron-induced cytotoxicity and placental damage, the iron chaperone poly-(rC)-binding protein 2 (PCBP2) sequesters intracellular iron. Nevertheless, the mechanistic details underlying PCBP2’s regulatory role in placental iron trafficking remain poorly characterized, underscoring the need for further research in this area [23].

### 2.3. Molecular Regulation of Placental Iron Homeostasis

Cellular iron metabolism during pregnancy is tightly regulated by the the coordinated action of Hepcidin-FPN axis and the iron regulatory protein–iron-responsive element (IRP-IRE) regulatory system to maintain iron homeostasis, ensuring adequate iron supply to the developing fetus. The hepcidin-FPN cycle serves as the central hub for systemic iron regulation, where maternal serum iron levels and hepcidin concentrations directly modulate the iron status of both the embryo and the placenta. Under conditions of elevated plasma or tissue iron stores, hepcidin binds to FPN, triggering its rapid ubiquitination and degradation [24]. This process limits cellular iron efflux, thereby reducing circulating iron levels. Conversely, during anemia states, transcriptional suppression of hepcidin antimicrobial peptide (HAMP) coupled with increased FPN expression facilitates enhanced iron mobilization from tissue stores into circulation, a critical adaptation to support the heightened erythropoietic demands of both mother and fetus.

During mid-to-late pregnancy, maternal hepcidin levels progressively decrease, enhancing iron export from enterocytes, hepatocytes, and macrophages to facilitate placental iron transport, though the underlying mechanisms remain unclear [10]. Studies have shown that maternal serum iron and hepcidin levels significantly influence embryonic and placental iron homeostasis. Iron-deficient mice exhibit reduced hepcidin expression, whereas iron-overloaded mice show elevated levels, suggesting that excess iron can partially counteract hepcidin suppression [25]. During pregnancy, both iron-deficient and iron-overloaded mice develop hypoferremia between E12.5 and E18.5. In iron-deficient pregnancies, this results from depleted iron stores, while in iron-overloaded conditions, elevated hepcidin levels restrict iron mobilization, thereby protecting the fetus from iron overload [25].

Under conditions of severe maternal iron deficiency, recent studies suggest that the placenta may prioritize its own iron requirements. In severely iron-deficient pregnant mice, FPN levels in the duodenum, placenta, and liver are significantly reduced, ultimately impairing iron transfer to the fetal circulation. In such situations, the transfer of iron into the plasma via FPN in the liver and splenic macrophages may serve as the dominant pathway for maternal–fetal iron transfer [26].

Intracellular iron levels are also tightly regulated by the IRP-IRE system. IRP1 and IRP2 modulate iron metabolism by binding to IREs in the untranslated regions (UTRs) of iron-related protein, thereby fine-tuning their translation based on cellular iron status. Under iron-deficient conditions, IRPs bind to the 5′-UTRs of *Fth1*, *Ftl*, *Hif2α*, and *Fpn* mRNAs, inhibiting their translation consequently limiting iron storage and export. Conversely, IRPs binding to the 3′-UTRs of *Tfr1* and *Dmt1* mRNAs stabilizes these transcripts and promotes iron uptake [25].

When iron levels are sufficient, IRPs undergo iron-dependent inactivation. Iron binding induces conformational changes that dissociate IRPs from target mRNAs, allowing for the regulated translation of iron metabolism genes to maintain cellular iron equilibrium [27]. Collectively, the IRP-IRE system dynamically balances iron uptake, storage, and export to ensure intracellular iron homeostasis.

## 3. Ferroptosis at the Maternal–Fetal Interface

### 3.1. Definition and Molecular Features of Ferroptosis

**Figure 1:**
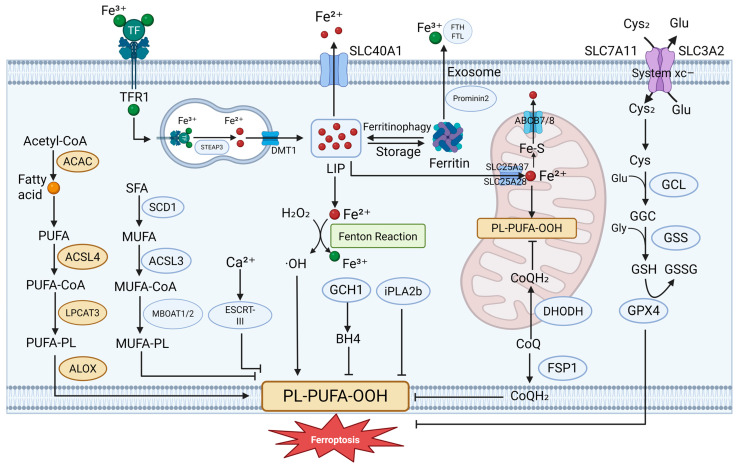
Molecular mechanisms of ferroptosis. Ferroptosis is regulated cell death driven by lipid peroxidation and redox imbalance. Multiple core pathways are involved: (1) the system Xc^−^–GSH–GPX4 axis defends against lipid peroxide accumulation; (2) iron overload promotes reactive oxygen species (ROS) production via the Fenton reaction, accelerating lipid damage; (3) FSP1 reduces CoQ to CoQH_2_ at the plasma membrane, limiting ferroptosis independently of GPX4; (4) DHODH functions in mitochondria to regenerate CoQH_2_ and protect mitochondrial integrity; and (5) additional modulators, such as the GCH1–BH4 axis and the ESCRT-III complex, influence ferroptotic sensitivity. These interconnected pathways form the molecular basis of ferroptosis regulation. ABCB7 ATP binding cassette subfamily B member 7, ACAC Acetyl-CoA carboxylase, ALOX lipoxygenase, CoQ coenzyme Q, Cys cysteine, Cys2 cystine, ESCRT-III Endosomal Sorting Complex Required for Transport III, GCH1 GTP Cyclohydrolase 1, GCL glutamate–cysteine ligase, GSH glutathione, GSSG oxidized glutathione, iPLA2b phospholipase A2 group VI, LIP labile iron pool, LPCAT3 lysophosphatidylcholine acyltransferase 3, MBOAT Membrane-Bound O-Acyltransferase, POR cytochrome P450 oxidoreductase, SCD1 stearoyl-CoA desaturase 1, SFA saturated fatty acid, SLC25A37, solute carrier family 25 member 37, SLC25A28 solute carrier family 25 member 28, SLC40A1 solute carrier family 40 member 1, TF transferrin, TFR1 transferrin receptor. Created in BioRender. Zhong, J. (2025) https://BioRender.com/zp0itls.

In 2012, Scott J. Dixon and colleagues at Columbia University defined “ferroptosis” as a distinct form of programmed cell death driven by excessive lipid peroxidation and characterized by iron dependency [28]. This form of cell death is distinct from well-characterized regulated cell death (RCD) pathways, such as apoptosis, pyroptosis, and necroptosis [29]. Morphologically, ferroptosis is distinguished by features including mitochondrial shrinkage, increased mitochondrial membrane density, and reduced mitochondrial cristae, which set it apart from other forms of cell death [28]. At the molecular level, ferroptosis is primarily defined by two key features: dysregulated iron accumulation and uncontrolled lipid peroxidation, as illustrated in Figure 1. Although diverse signaling pathways can initiate ferroptosis, they converge on the disruption of cellular antioxidant defenses, particularly through the direct or indirect inhibition of glutathione peroxidases (GPXs). This inhibition leads to increased lipid peroxidation and the generation of ROS, which collectively drive the process of ferroptotic cell death [30]. GPX4 serves as the central guardian against ferroptosis by catalyzing the reduction of phospholipid hydroperoxides (PLOOHs) to nontoxic alcohols, thereby terminating the lipid peroxidation chain reactions. However, when GPX4 function is compromised or intracellular glutathione (GSH) levels are depleted, the resulting accumulation of lipid peroxides induces ferroptosis [31]. Beyond GPX4, multiple parallel pathways regulate ferroptosis susceptibility, underscoring the complex interplay between iron metabolism, redox homeostasis, and cellular defense mechanisms. For example, the ferroptosis suppressor protein 1 (FSP1)- coenzyme Q10 (CoQ10) pathway serves as a major suppressor of ferroptosis by CoQ10 to its antioxidant form, thereby interrupting the lipid peroxidation process [32]. Additionally, the tetrahydrobiopterin (BH4) and its precursor dihydrobiopterin (BH2) pathway, functions both as a radical-trapping antioxidant and as a supporter of CoQ10 biosynthesis, synergistically enhancing cellular resistance to oxidative stress and preventing ferroptosis [33].

Moreover, accumulating evidence indicates that ferroptosis is orchestrated by multiple cellular compartments and metabolic circuits beyond the cytosol. Mitochondria, the endoplasmic reticulum, peroxisomes, and lipid droplets all participate in modulating susceptibility to ferroptosis by influencing iron handling, lipid metabolism, and redox balance [34]. For example, mitochondrial metabolic pathways can contribute precursors of lipid peroxides, while peroxisomal lipid synthesis and remodeling pathways regulate the composition of polyunsaturated fatty acids incorporated into membrane phospholipids, thereby shaping ferroptotic vulnerability. Disruption of ion transport across the plasma membrane has also been proposed as a downstream execution mechanism, as lipid peroxidation alters membrane permeability and ionic gradients [34].

Iron metabolism is also critical in regulating ferroptosis, as iron catalyzes the Fenton reaction, generating highly reactive ROS that drive lipid peroxidation. The susceptibility of cells to ferroptosis is directly influenced by labile iron pool dynamics, with regulatory proteins such as ferritin (iron storage) and transferrin (iron uptake) receptor playing essential roles in maintaining iron homeostasis [35]. Thus, the cellular redox balance, particularly the availability of GSH and the activity of GPX4, constitutes a fundamental regulatory axis regulating ferroptosis. Notably, transcriptional master regulators, including nuclear factor erythroid 2–related factor 2 (NRF2) and other cap ‘n’ collar (CNC) family transcription factors, coordinate gene expression programs that integrate signals from different organelles and pathways to control ferroptosis globally [36]. This highly context-dependent nature of ferroptosis explains why its regulation varies across different cell types and death-inducing conditions.

The pathophysiological relevance of ferroptosis extends across diverse disease states, including neurodegenerative diseases, ischemia-reperfusion injury, and cancer. Notably, in the context of the maternal–fetal interface, ferroptosis may critically contribute to pregnancy-related complications such as preeclampsia by impairing placental cell function and viability.

Therefore, understanding the molecular mechanisms of ferroptosis and its impact on the maternal–fetal interface is essential for developing targeted therapies to mitigate these pregnancy-related complications.

### 3.2. Ferroptosis in Preeclampsia and Other Pregnancy Disorders

Ferroptosis, an iron-dependent form of regulated cell death, is a critical process at the maternal–fetal interface, a vital zone where maternal and fetal tissues interact [37,38]. This interface mediates critical physiological processes, including nutrient exchange, waste elimination, and immune tolerance during pregnancy. Dysregulation of iron homeostasis and oxidative stress at the maternal–fetal interface may induce ferroptosis, potentially contributing to pregnancy complications such as preeclampsia and intrauterine growth restriction (IUGR).

Trophoblast cells, as the key components of the placenta, are particularly susceptible to ferroptosis owing to their unique physiological characteristics. and exposure to fluctuating oxygen levels, which exacerbate oxidative stress. The accumulation of iron and the subsequent generation of ROS in these cells can trigger lipid peroxidation, a hallmark of ferroptosis. If antioxidant defenses, particularly GPX4, fail to control this process, the resulting lipid peroxides can damage cellular membranes, leading to cell death [17].

Growing clinical and experimental evidence suggests that disturbances in iron homeostasis are strongly associated with the pathogenesis of PE, particularly through their impact on the maternal–fetal interface. In normal pregnancy, systemic and placental iron metabolism are tightly regulated to support fetal development while minimizing oxidative stress. However, in PE, this balance is frequently compromised. Proper placental iron transport and maternal iron levels are critical for both maternal and fetal health. While iron supplementation can prevent maternal anemia, excessive iron intake may trigger ferroptosis and pose additional risks. A randomized, placebo-controlled trial involving 723 pregnant women with mid-pregnancy hemoglobin levels > 13.2 g/dL assigned participants to either daily ferrous sulfate (150 mg, containing 50 mg of elemental iron) or a placebo [39]. The results showed that long-term ferrous sulfate use was associated with higher rates of low birth weight and hypertension, suggesting that routine iron supplementation may not be beneficial for non-anemic pregnant women. Chronic daily iron intake exceeding the tolerable upper limit (45 mg/day) can lead to iron overload and hemochromatosis [40]. Although rare, studies indicate that NTBI might aid in recovering from iron deficiency, but excessive dietary iron intake during pregnancy could induce placental oxidative stress, warranting further investigation [41]. Disruption of iron homeostasis in the placenta can lead to ferroptosis in trophoblasts, impairing placental function and causing various pregnancy complications. Emerging evidence highlights strong association between preeclampsia, iron overload, and ferroptosis.

The occurrence of ferroptosis at the maternal–fetal interface is not solely a pathological event; it may also play a role in normal placental development by regulating trophoblast turnover and maintaining tissue homeostasis. However, excessive or inappropriate activation of ferroptosis can disrupt placental function, impairing nutrient and oxygen delivery to the fetus, underscoring the critical need for tightly regulated iron metabolism and redox balance in this unique environment.

Recent studies have highlighted the susceptibility of placental trophoblasts to ferroptosis and provided evidence linking ferroptosis to placental dysfunction, which is implicated in various pregnancy-related disorders. In preeclampsia, abnormal placental development leads to placental ischemia and hypoxia, which are considered the primary drivers of excessive ROS production (72) [42]. Intracellular iron accumulation is strongly associated with ferroptosis, as iron generates excessive ROS through the Fenton reaction and increases lipoxygenase (LOX) activity, leading to lipid peroxidation [38]. These oxidative insults can impair trophoblast proliferation, migration, and invasion, ultimately compromising placental vascular development and function. Moreover, iron-induced oxidative stress may activate hypoxia-inducible factors (e.g., HIF-1α) and pro-inflammatory pathways (e.g., nuclear factor kappa B (NF-κB)), further exacerbating endothelial dysfunction and contributing to systemic hypertension [43,44,45]. PE patients exhibit significantly elevated serum iron levels, placental ferritin concentration, transferrin saturation, and hepcidin levels compared to healthy controls [42,46]. Histological analyses of PE placental tissues further support these observations. Perl’s Prussian blue staining, a classical method for detecting ferric iron (Fe^3+^) deposits, reveals iron accumulation within the syncytiotrophoblasts, stromal macrophages, and vascular endothelium [47,48]. In some cases, enhanced Perl’s or Turnbull’s staining has been used to detect even low-level iron deposition, confirming the presence of both ferric (Fe^3+^) and ferrous (Fe^2+^) iron species [49]. PE and eclampsia patients, demonstrate markedly increased levels of malondialdehyde (MDA), a lipid peroxidation product, along with decreased levels of antioxidant enzymes such as GPX4, glutathione reductase, and catalase [50]. The ferroptosis-related molecule acyl-CoA synthetase long-chain family member 4 (ACSL4) is upregulated in of PE placental tissues and in hypoxia-exposed trophoblasts [51]. One study identified relatively high concentrations of polyunsaturated fatty acids, including docosapentaenoic acid (DPA), arachidonic acid, and linoleate, in the mitochondria of severe preeclampsia patients, implicating ferroptosis in placental dysfunction [52]. The combined evidence—elevated MDA, depleted GPX4, altered metabolism of Polyunsaturated Fatty Acids (PUFA), and iron overload—strongly supports the role of ferroptosis in the pathogenesis of preeclampsia.

At the genetic level, emerging evidence suggests that ferroptosis may contribute to the pathogenesis of early-onset preeclampsia. Comparative analysis of ferroptosis-related gene expression profiles in placental samples revealed distinct patterns between early-onset and late-onset PE, with differentially expressed ferroptosis-related genes (FRGs) associated with early-onset cases. These genes were notably enriched in pathways related to hypoxia and iron homeostasis, suggesting a unique molecular signature in early-onset disease [53].

Recent transcriptomic analyses, including single-cell RNA sequencing (scRNA-seq) of placental tissues, have provided additional mechanistic insight into placental iron dysregulation in PE. In PE placentas, upregulation of key iron metabolism genes such as TFR1 and FTH1 has been observed, particularly in trophoblast subsets and immune cell populations [54,55]. Interestingly, recent developmental studies have demonstrated that iron levels are not only linked to oxidative stress but also critically affect the epigenetic regulation of essential embryonic genes such as sex-determining region Y (Sry) For example, ferrous iron (Fe^2+^) availability is necessary for lysine demethylase 3A (KDM3A)-dependent histone demethylation required for Sry activation during male sex determination, and iron deprivation can lead to sex reversal phenotypes. Although KDM3A itself has not been systematically studied in the context of preeclampsia, evidence suggests that epigenetic regulation, including DNA and histone methylation in the placenta, may play a role in disease pathogenesis [56]. These findings imply that maternal iron overload or disturbed iron homeostasis in PE could impact not only placental function and oxidative injury but also broader aspects of fetal growth and differentiation through iron-dependent epigenetic mechanisms.

Similar pathological mechanisms are observed in IUGR, where altered iron metabolism and oxidative stress within the placenta induces ferroptosis, reducing the placenta’s capacity to support fetal growth and leading to restricted fetal development [57]. This underscores the critical need for tightly regulating ferroptosis to maintain adequate placental function and fetal health. Furthermore, gestational diabetes mellitus (GDM) is one of the most common pregnancy complications, and elevated iron levels have been identified as a risk factor [58]. While some studies suggest that a high dietary intake of heme iron before and during early pregnancy may increase the risk of GDM, this association remains controversial. GDM patients typically demonstrate higher iron stores compared to healthy controls, and the hyperglycemia characteristic of GDM has been shown to impair trophoblast function and induce ferroptosis in placental cells [59,60].

These insights into the relationship between ferroptosis and maternal–fetal diseases emphasize the need for further research to elucidate the underlying mechanisms and explore potential therapeutic interventions targeting ferroptosis. Such strategies could improve placental health and pregnancy outcomes by modulating iron metabolism and oxidative stress at the maternal–fetal interface. Additionally, the practice of routine iron supplementation during pregnancy warrants reconsideration, given these significant challenges.

## 4. Macrophages at the Maternal–Fetal Interface

### 4.1. Origin, Distribution, and Polarization of Macrophages in Pregnancy

Macrophages are versatile immune cells that play essential roles in maintaining tissue homeostasis, facilitating repair and remodeling, and defending against pathogens during pregnancy [61]. Traditionally, it was believed that macrophages mainly originated from circulating blood monocytes derived from myeloid progenitors in the bone marrow [62]. However, accumulating evidence has demonstrated that some macrophage populations arise independently of hematopoietic stem cells (HSCs). Specifically, a subset of macrophages originates from yolk sac progenitors during early embryogenesis, where they differentiate locally into yolk sac macrophages. These cells subsequently migrate via the circulation to embryonic tissues and mature into tissue-resident macrophages. Another subset emerges later from erythromyeloid progenitors (EMPs) derived from the yolk sac endothelium and from HSCs in the aorta-gonad-mesonephros (AGM) region. These progenitors differentiate into fetal monocytes in the fetal liver and later develop into adult tissue-resident macrophages [63].

Macrophage polarization describes the process by which macrophages acquire distinct functional phenotypes in response to diverse microenvironmental signals, including microbial components, cellular debris, and activated lymphocytes 19 July 2025 9:49:00 AM. Generally, macrophages are classified into two major subsets based on their activation profiles, surface markers, cytokine secretion patterns, and biological functions: classically activated or pro-inflammatory (M1) macrophages, and alternatively activated or anti-inflammatory (M2) macrophages [61]. This remarkable plasticity enables macrophages to adapt to different physiological and pathological conditions during pregnancy. M1 macrophages are characterized by their strong pro-inflammatory and antimicrobial activities. They can be induced by signals such as lipopolysaccharide (LPS), interferon-gamma (IFN-γ), TNF-α, granulocyte-macrophage colony-stimulating factor (GM-CSF), and Toll-like receptor (TLR) ligands, and express markers including TLR-2, TLR-4, CD80, CD86, inducible nitric oxide synthase (iNOS), and major histocompatibility complex class II (MHC-II). M1 macrophages produce cytokines and chemokines such as TNF-α, IL-1α, IL-1β, IL-12, C-X-C motif chemokine ligand 9 (CXCL9), and CXCL10, thereby amplifying inflammation through positive feedback loops. This phenotype is regulated by transcription factors such as signal transducer and activator of transcription 1 (STAT1), STAT5, NF-κB, interferon regulatory factor 3 (IRF3), and IRF5 [64,65,66,67,68]. In contrast, M2 macrophages are induced by IL-4, IL-13, IL-10, M-CSF, and other factors, and express surface markers including CD206, CD163, CD209, mannose receptor, found in inflammatory zone 1 (FIZZ1), and chitinase-like proteins Ym1 and Ym2 (Ym1/2) [69,70]. They secrete cytokines and chemokines such as IL-10, transforming growth factor-beta (TGF-β), C-C motif chemokine ligand 1 (CCL1), CCL17, CCL18, CCL22, and CCL24, which contribute to anti-inflammatory responses and tissue remodeling [71]. Transcription factors like STAT6, IRF4, jumonji domain containing 3 (JMJD3), peroxisome proliferator-activated receptor delta (PPARδ), and PPARγ are involved in regulating M2-associated gene expression [72]. Additionally, M2 macrophages are further divided into subtypes—M2a, M2b, M2c, and M2d—each with distinct functions. M2a macrophages, induced by IL-4 and IL-13, produce IL-10, TGF-β, and other mediators involved in tissue repair [73]. M2b macrophages are activated by immune complexes and microbial stimuli and secrete both pro-inflammatory and anti-inflammatory cytokines, thereby modulating immune responses [74]. M2c macrophages, induced by glucocorticoids and IL-10, participate in the clearance of apoptotic cells [75]. M2d macrophages, also referred to as tumor-associated macrophages, are driven by Toll-like receptor ligands, IL-6, and adenosine receptor activation, and contribute to angiogenesis and tumor progression by releasing TGF-β, IL-10, and vascular endothelial growth factor (VEGF) [76].

### 4.2. Functions and Phenotypic Dynamics of Decidual Macrophages in Normal and Pathological Pregnancy

At the maternal–fetal interface, the decidua contains diverse populations of maternal-derived innate immune cells, including decidual natural killer (dNK) cells, innate lymphoid cells (dILCs), placenta-associated macrophages and monocytes (PAMMs), and decidual dendritic cells (dDCs) [77]. Among these, dNK cells are the most abundant, comprising approximately 70% of decidual immune cells, while decidual macrophages (DMs) represent the second largest group, accounting for about 20–25% [78]. DMs display a unique transcriptional profile and phenotypic diversity compared to macrophages in other tissues, reflecting their specialized roles in pregnancy. Early studies proposed subclassification of DMs into CD11c high and CD11c low subsets, distinguished by differential expression of CD206 and CD209, and associated with functions ranging from lipid metabolism and inflammation to extracellular matrix formation and tissue growth [79,80]. Further stratification based on C-C chemokine receptor type 2 (CCR2) and CD11c expression has identified subsets with either pro-inflammatory or anti-inflammatory features [81]. Additionally, CD14+/CD16+ macrophages exhibiting immunoregulatory gene signatures characteristic of M2-like phenotypes have been implicated in trophoblast invasion, placental development, and uterine vascular remodeling [82].

During normal pregnancy, macrophage polarization dynamically shifts in response to gestational stage and microenvironmental cues. In the peri-implantation phase, macrophages predominantly adopt an M1 phenotype that promotes embryo adhesion to the decidua. As trophoblasts invade the maternal endometrium, macrophages progressively transition toward a mixed M1/M2 state, supporting extensive spiral artery remodeling and angiogenesis critical for placental perfusion [65,83]. Following placental maturation, M2 polarization becomes predominant, contributing to immune tolerance and maintenance of pregnancy [65,84]. Decidual macrophages also participate in apoptotic cell clearance, thereby preventing the release of paternal antigens, facilitating trophoblast invasion, and sustaining a stable immune microenvironment [68,85]. Through the secretion of cytokines such as IL-1β, TNF-α, and TGF-β, macrophages coordinate trophoblast migration and extracellular matrix remodeling by regulating matrix metalloproteases (MMP-2, MMP-3, MMP-9) and urokinase plasminogen activators [86]. Moreover, interactions between dNK cells and macrophages modulate trophoblast invasion: macrophages can produce TGF-β to suppress NK cell cytotoxicity, whereas dNK-derived GM-CSF promotes M1 polarization and trophoblast infiltration [87].

In pregnancy complications such as preeclampsia (PE) and intrauterine growth restriction (IUGR), this finely tuned balance of macrophage polarization is disrupted. A shift toward an increased M1 population and decreased M2 macrophages leads to an inflammatory microenvironment within the placenta [88]. Excessive secretion of TNF-α and IFN-γ by activated macrophages induces trophoblast apoptosis and restricts their invasive capacity [89]. Impaired spiral artery remodeling, a hallmark of PE, compromises uteroplacental blood flow and contributes to fetal growth restriction and placental abruption [90]. Decidual macrophages are key contributors to vascular remodeling through the production of VEGF, placental growth factor (PIGF), and MMPs [91,92]. Factors such as pregnancy-specific glycoproteins and protein kinase C (PKC) signaling modulate VEGF pathways and impact macrophage-mediated angiogenesis [93,94,95]. Additionally, alterations in the maternal gut microbiota and reduced short-chain fatty acid production exacerbate inflammation and favor M1 polarization, further contributing to placental dysfunction [96]. Excess TNF-α produced by macrophages also promotes apoptosis by upregulating X-linked inhibitor of apoptosis protein (XIAP), thereby impairing trophoblast invasion and survival [97,98]. Collectively, these findings highlight the essential role of macrophage plasticity in normal gestation and its dysregulation in pregnancy-related disorders, suggesting that targeting macrophage polarization may offer promising therapeutic strategies.

## 5. Interactions Between Iron and Macrophages at the Maternal–Fetal Interface

### 5.1. Iron as a Modulator of Macrophage Polarization and Function

Macrophages regulate iron metabolism, and conversely, iron homeostasis profoundly affects their functional phenotypes. Various genes and enzymes involved in heme processing—including nicotinamide adenine dinucleotide phosphate (NADPH) oxidase (NOX2), cyclooxygenase (COX), heme dioxygenases (IDO1 and IDO2), cystathionine β-synthase (CBS), nitric oxide synthases (NOS), and iron-responsive prolyl hydroxylase (PHD2)—are important modulators of macrophage activities in the context of iron balance [99].

Emerging evidence highlights a dynamic interplay between iron homeostasis and NF-κB signaling in regulating macrophage polarization. Both iron overload and deficiency can activate NF-κB, albeit through distinct upstream cues, leading to increased expression of pro-inflammatory mediators such as TNF-α, iNOS, and MMP-9, characteristic of M1 polarization [100,101,102]. For instance, Fe^2+^ directly stimulates NF-κB via Inhibitor of κB kinase (IKK) activation in Kupffer cells [103], while iron deficiency enhances inflammation via the p38 mitogen-activated protein kinase (MAPK)–NF-κB axis in foam cells [101]. Iron status also affects ferroportin transcription through NF-κB-dependent recruitment of histone deacetylases (HDACs 1/3), linking inflammation to intracellular iron retention [104]. Moreover, NF-κB regulates Spic, a transcription factor that promotes iron efflux and limits inflammation, while IFN-γ counteracts this mechanism through STAT1 signaling, fine-tuning macrophage phenotype under different immune contexts [105].

Interestingly, the effect of iron on NF-κB is highly context-dependent: while cytosolic Fe^2+^ can activate pro-inflammatory signaling, lysosomal Fe^2+^ release via the transient receptor potential mucolipin 1 (TRPML1) channel promotes prolyl hydroxylase (PHD) activation, which in turn suppresses NF-κB and IL-1β transcription [106]. Likewise, elevated intracellular iron under non-infectious conditions may inhibit NF-κB nuclear translocation and favor M2 polarization [107].

Notably, iron exerts a biphasic regulatory effect on macrophage function. On one hand, it supports the activity of hemoproteins such as iNOS and NOX2, enhancing STAT1-dependent nitric oxide production and M1-type antimicrobial responses. On the other hand, iron accumulation suppresses STAT1/3 activation, downregulates iNOS transcription, and promotes anti-inflammatory pathways involving HO-1, IL-10, and STAT3 [105,108]. This duality positions iron as a central rheostat balancing macrophage polarization between inflammatory and regulatory states, shaped by its localization, redox activity, and the surrounding cytokine milieu.

In addition to modulating NF-κB activity, iron overload promotes M1 macrophage polarization through several alternative mechanisms, including inflammasome activation, MAPK-dependent cytokine stabilization, redox-sensitive transcriptional pathways, and metabolic shifts. One important route involves the activation of the NOD-like receptor family pyrin domain-containing 3 (NLRP3) inflammasome by intracellular labile iron. Elevated Fe^2+^ levels initiate mitochondrial dysfunction, lysosomal destabilization, and ROS accumulation, collectively triggering NLRP3-dependent caspase-1 activation and IL-1β maturation [109]. This mechanism highlights how iron functions not merely as a nutrient or damage signal, but as a direct pro-inflammatory trigger that enhances M1 polarization through innate immune sensors.

In the context of spinal cord injury, iron overload sustains inflammatory polarization via the p38 MAPK–MAPK-activated protein kinase 2 (MK2) axis [110]. Phosphorylated MK2 stabilizes TNF expression by inactivating tristetraprolin (TTP), an mRNA decay-promoting protein. By preventing degradation of TNF transcripts, this pathway prolongs the inflammatory state of macrophages despite signals that typically promote M2 transition, such as phagocytosis. Loss of MK2 function reduces TNF production and restores M2 conversion, underscoring the crucial role of post-transcriptional regulation in iron-dependent macrophage fate decisions.

Iron-induced ROS generation also activates the acetyl-p53 signaling pathway, which contributes to M1 polarization through transcriptional reprogramming [111]. Oxidative stress increases the activity of p300/CREB-binding protein (CBP) acetyltransferases, leading to p53 acetylation and upregulation of p21 and other inflammatory effectors. Inhibition of ROS or p53 acetylation effectively reverses M1 polarization, revealing that iron-sensitive redox signaling governs not only stress responses but also macrophage functional identity.

Finally, iron shapes macrophage polarization by influencing cellular metabolism. Excess iron enhances aerobic glycolysis, as evidenced by elevated extracellular acidification rates without changes in oxidative respiration [112]. This metabolic shift, characteristic of classically activated macrophages, provides the energy and biosynthetic support necessary for sustained cytokine production and inflammatory activation. In disease contexts such as atherosclerosis, this iron-driven metabolic reprogramming intensifies macrophage-mediated inflammation and accelerates lesion development.

Together, these findings illustrate that iron orchestrates macrophage polarization through diverse, interlinked pathways that extend beyond canonical NF-κB signaling. By targeting mRNA stability, metabolic flux, inflammasome priming, and transcriptional regulation, iron establishes a multilayered control network that locks macrophages into a pro-inflammatory state under pathological conditions.

Moreover, iron overload and lipid peroxidation can induce ferroptosis, a distinct form of iron-dependent cell death. During ferroptosis, affected cells release damage-associated molecular patterns (DAMPs), including oxidized phospholipids, 4-hydroxynonenal (4-HNE), high-mobility group box 1 (HMGB1), prostaglandin E2 (PGE2), DNA, and ATP, which recruit and activate macrophages [113,114,115,116]. For example, oxidized phospholipids and 4-HNE act as “eat me” signals via TLR2 recognition, while HMGB1 engages receptor for advanced glycation end-products (RAGE) receptors and triggers NF-κB signaling, promoting inflammation and reinforcing M1 polarization [114,115]. Ferroptosis-derived mediators such as PGE2 and 8-hydroxy-2′-deoxyguanosine (8-OHdG) further amplify inflammatory responses and chemokine production [116]. Together, these findings illustrate that iron metabolism critically modulates macrophage function by integrating metabolic, transcriptional, and cell death pathways.

### 5.2. Macrophage-Mediated Regulation of Iron Sequestration and Availability

Macrophages serve as central regulators of iron homeostasis through two main primary mechanisms. First, within the “erythroblastic island,” erythroblasts differentiate into reticulocytes around a central “nurse macrophage.” This macrophage provide developing erythroid cells with iron, heme, and other essential factors to support erythropoiesis [117]. Second, macrophages acquire iron primarily through extracellular iron (Fe^3+^) bound to TFR1 and through erythrophagocytosis (EP), where they engulf and degrade aging or damaged red blood cells. This process is mainly mediated by splenic red pulp macrophages (RPMs) and bone marrow macrophages (BMMs), driven by the heme-regulated transcription factor SPI-C [118]. Within macrophages, engulfed red blood cells are degraded in lysosomes, releasing heme from hemoglobin. Heme is then transported into the cytoplasm by heme-responsive gene-1 (HRG-1) and degraded by HO-1, releasing iron from the heme’s protoporphyrin ring. This iron can either be exported from the macrophage via FPN or stored intracellularly in FTN (ferritin). Heme can also be exported out of the cell via FLVCR1 (Feline Leukemia Virus Cellular Receptor 1). Once exported, iron binds to transferrin, enters the bloodstream, and is absorbed by nucleated erythroid cells through their transferrin receptors. In these cells’ mitochondria, ferrochelatase (FECH) catalyzes the insertion of iron into protoporphyrin IX (PPIX) to re-form heme [99,117].

**Figure 2:**
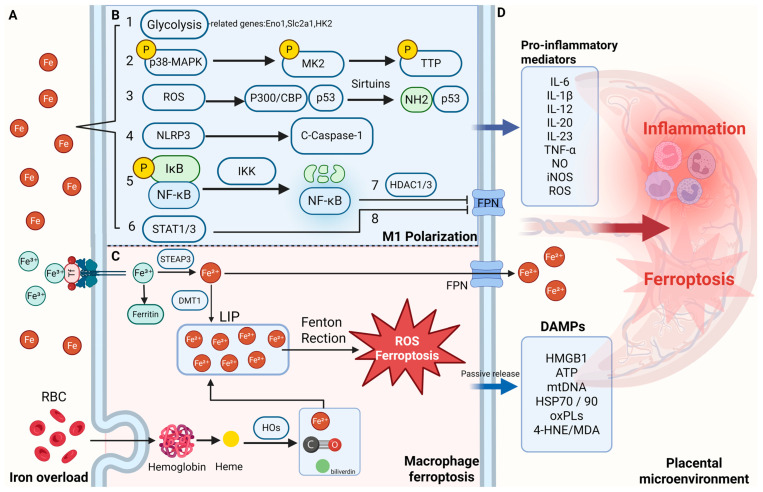
Iron overload–driven macrophage activation and ferroptosis in the placental microenvironment. (**A**) Iron accumulation in the maternal–fetal interface under pathological conditions. (**B**) Mechanisms by which iron overload promotes macrophage M1 polarization. Iron overload drives M1 polarization of macrophages through multiple pathways: (1) by enhancing glycolysis and upregulating genes such as Eno1, Slc2a1, and HK2, it promotes metabolic reprogramming and pro-inflammatory cytokine release; (2) excessive iron activates the MK2 pathway, inducing TNF expression and promoting the shift from M2 to M1, resulting in sustained inflammatory phenotypes; (3) iron-induced ROS accumulation activates the acetylated p53 axis, further reinforcing pro-inflammatory polarization; (4) labile Fe^2+^ directly activates the NLRP3 inflammasome and caspase-1, promoting IL-1β maturation and amplifying NF-κB/M1 signaling; (5) through Fenton chemistry, Fe^2+^ generates hydroxyl radicals (•OH) that activate IKK, degrade IκBα, and promote NF-κB nuclear translocation and TNF-α transcription; (6) iron chelation (e.g., DIBI) reduces Fe^2+^ levels, suppresses STAT1/3 activation, and lowers IL-6, IL-1β, and NO production, exerting anti-inflammatory effects; (7) inflammation-driven NF-κB activation induces HDAC1/3, which represses Slc40a1 (ferroportin) expression, leading to intracellular iron retention in macrophages; and (8) inflammatory cytokines upregulate hepcidin via STAT3 signaling, further inhibiting ferroportin-mediated iron export and exacerbating iron sequestration within macrophages. (**C**) Iron uptake and ferroptosis in macrophages. Macrophages acquire iron through two major pathways: (1) uptake of transferrin-bound ferric iron (Fe^3+^) via transferrin receptor 1 (TFR1); and (2) erythrophagocytosis (EP), a process in which aged or damaged red blood cells are engulfed and degraded. The imported iron contributes to the labile iron pool (LIP), which can catalyze Fenton reactions, generating hydroxyl radicals (•OH) that initiate lipid peroxidation and ferroptosis. During ferroptosis, intracellular iron is partially exported through ferroportin (FPN), while damage-associated molecular patterns (DAMPs) are passively released into the extracellular space, amplifying inflammation. (**D**) Inflammatory cytokines and DAMPs amplify placental inflammation and ferroptosis. 4-HNE 4-hydroxynonenal, C-Caspase-1 cleaved caspase-1, CoQ coenzyme Q, DAMPs damage-associated molecular patterns, HDAC1/3 histone deacetylase 1/3, HOs heme oxygenases, IKK IκB kinase, LIP labile iron pool, MDA malondialdehyde, MK2 MAPK-activated protein kinase 2, NLRP3 NOD-like receptor family pyrin domain containing 3, NF-κB nuclear factor kappa-light-chain-enhancer of activated B cells, oxPLs oxidized phospholipids, P300/CBP P300/CREB-binding protein, p38-MAPK p38 mitogen-activated protein kinase, STAT1/3 signal transducer and activator of transcription 1/3, TTP Tristetraprolin. Created in BioRender. Zhong, J. (2025) https://BioRender.com/dr3egzh.

Disruption of these finely tuned processes—especially the phagocytosis of damaged or aging red blood cells by macrophages—can lead to excessive iron release, potentially causing cytotoxic effects and ferroptosis, as illustrated in Figure 2.

Macrophages employ multiple iron accumulation mechanisms, thereby limiting microbial infections. M1 macrophages are known for their pro-inflammatory and antimicrobial functions, which include secreting high levels of cytokines for tissue repair [119]. Iron accumulation is another key mechanism by which M1 macrophages exert these effects [120]. Both mammals and microbes have evolved strategies to compete for iron, a critical resource for pathogen growth and DNA replication. In response, the host regulates iron metabolism through mechanisms mediated by macrophage-produced inflammatory cytokines, competing for iron and preventing pathogens from acquiring it.

During acute inflammation, macrophages release cytokines that induce the secretion of iron-binding proteins and lactoferrin in the liver and secrete hepcidin, which downregulates FPN, leading to iron retention within macrophages and Kupffer cells [119,120,121]. This restricts iron availability for erythropoiesis. Additionally, studies have shown that adding ferroptosis inducers to macrophages can enhance bacterial suppression, suggesting ferroptosis as a potential pathway for macrophages to inhibit intracellular pathogens [122]. However, excessive iron accumulation in the reticuloendothelial system can lead to chronic inflammatory anemia and tissue damage, highlighting the importance of maintaining iron homeostasis for macrophage immune function.

The expression profiles of iron-related genes differ substantially between M1 and M2 macrophages, primarily due to the “iron accumulation” mechanism characteristic of M1 macrophages [99,119]. M1 macrophages consistently exhibit elevated expression of HAMP (encoding hepcidin), FtH/FtL (ferritin), while showing reduced expression of FPN, IRP1/2, and TFR1. Conversely, M2 macrophages exhibit increased expression of FPN, HO-1, and TFR1, coupled with decreased FTN levels [123,124,125]. During inflammatory conditions these gene expression patterns become more pronounced, leading to intracellular iron accumulation, which activates the pro-inflammatory functions of M1 macrophages.

### 5.3. Pro-Inflammatory Mediators and ROS from M1 Macrophages Induce Ferroptosis

The mitochondrial inner membrane respiratory chain is one of the primary sites of ROS production. During electron transfer to oxygen, partial oxygen production generates various ROS, including hydrogen peroxide (H_2_O_2_), superoxide anion (O^2−^), and hydroxyl radical (OH^−^) [126]. Another major source of ROS emerges during signal transduction involving growth factors, where NADPH oxidases (NOX1–5 and dual oxidase (Duox1–2)) reduce oxygen to O^2−^. The NADPH oxidase family members are expressed on the plasma membranes, with NOX1, NOX2, and NOX4 demonstrating particularly high activity in macrophages [127]. Beyond these primary sources, ROS production also occurs in the endoplasmic reticulum during protein folding, mediated by enzymes such as monoamine oxidase, xanthine oxidase, cytochrome P450, cyclooxygenase, as well as in peroxisomes and through protein 66 Src homology 2 domain-containing (P66shc) activity [128].

Macrophage-generated ROS are essential for killing pathogens, contributing to both innate and adaptive immunity. However, excessive ROS production can lead to oxidative stress and cytotoxicity, ultimately triggering ferroptosis, an iron-dependent form of regulated cell death. Additionally, cytokines produced by macrophages or induced by ROS can regulate systemic iron homeostasis, with some cytokines promoting and others inhibiting ferroptosis.

During ferroptosis induction, excessive intracellular free iron partitions into two pathways: a portion enters the mitochondria, contributing to oxidative stress, while the remainder generates substantial ROS through the Fenton reaction. To counteract this process, cells have evolved several endogenous ferroptosis inhibitory systems, including the cyst(e)ine/GSH/GPX4 system, NADPH/FSP1/CoQ10 system, GTP cyclohydrolase 1 (GCH1)/BH4/dihydrofolate reductase (DHFR) system, and GPX4/dihydroorotate dehydrogenase (DHODH) system. The cyst(e)ine/GSH/GPX4 system, for instance, regulates ROS levels via the Nrf2 pathway, and its depletion result in polyunsaturated fatty acids peroxidation, triggering ferroptosis.

Research has shown that high levels of ROS are secreted by myometrial cells during preterm labor, with approximately 90% of this ROS originating from infiltrating macrophages [129,130]. The ROS released by macrophages also induce the expression of uterine labor markers. Additionally, changes in ferroptosis-related markers have been observed in trophoblast cells from the placentas of preterm birth patients, suggesting a link between preterm birth and ferroptosis. This indicates that ROS and cytokines secreted by macrophages at the maternal–fetal interface may mediate trophoblast ferroptosis, leading to placental dysfunction and related pregnancy complications.

Further studies have highlighted the connection between macrophages and ferroptosis in other parts of the body. For example, monocyte-derived macrophages (MoMCs) secrete neutrophil cytosolic factor 1 (NCF1), which induces ROS production and oxidizes phospholipids, activating TRL4-dependent hepcidin in hepatocytes. This process promotes Kupffer cell ferroptosis and exacerbates metabolic-associated steatohepatitis (MASH) progression [131].

In addition to NCF1, various cytokines secreted by macrophages regulate their functions and the surrounding microenvironment, with ferroptosis being one of the influenced processes. For instance, the M1 macrophage marker IL-6 can regulate hepcidin transcription via the Janus kinase (JAK)-STAT3 pathway, affecting iron homeostasis and leading to ferroptosis. Another M1 marker, TNF-α, upregulates ACSL3 expression, causing intracellular lipid accumulation [132]. Similarly, IL-1β, like IL-6 and TNF-α, is an inflammatory cytokine secreted by macrophages, and it can promote the expression of ferroptosis markers [133]. IL-1β also induces FPN expression through the MAP kinase pathway and activates p38 MAPK, leading to intracellular iron depletion [134].

## 6. Iron Overload and Macrophage Dysfunction in Preeclampsia

Recent advances have underscored the iron–immune axis as a central contributor to trophoblast dysfunction, oxidative stress, and systemic inflammation in preeclampsia (PE). Dysregulated iron metabolism and ferroptosis converge to drive placental injury and vascular maladaptation, providing multiple therapeutic entry points to restore homeostasis.

### 6.1. Iron-Responsive Immune Pathways and Their Association with Disease Severity

Several iron-regulated immune pathways exhibit dysregulation in PE with the degree of dysfunction correlating with disease severity. HO-1 is a pivotal iron-responsive enzyme that exerts multiple functions in the pathogenesis of preeclampsia (PE). HO-1 catalyzes the degradation of heme into biliverdin, carbon monoxide (CO), and free iron, thereby conferring potent antioxidant, anti-inflammatory, and vasoprotective effects. Genetic polymorphisms that diminish HO-1 expression, such as longer guanine–thymine (GTn) repeats within the heme oxygenase 1 gene (HMOX1) promoter region, have been strongly linked to an increased risk of PE, particularly in severe and early-onset forms [135]. Specifically, longer maternal GT repeats are associated with late-onset PE and reduced HO-1 levels, while extended fetal repeats correlate with more pronounced disease phenotypes and decreased HO-1 expression in both placental tissue and maternal serum [135].

Nevertheless, investigations of HO-1 expression in PE have produced inconsistent results. Several studies have reported attenuated HO-1 activity in affected patients, as evidenced by lower exhaled CO concentrations and compromised antioxidant capacity, suggesting impaired cytoprotective function [136]. Recent work has also demonstrated reduced expression of cluster of differentiation 151 (CD151) and HO-1 in placentas from women with PE compared to controls, indicating that CD151 may promote HO-1 expression and could represent a potential therapeutic target [137]. Additionally, emerging evidence has shown that functional impairment of mesenchymal or stromal cells isolated from PE placentas may contribute to disease progression, with HO-1 deficiency playing a key role in this process [138]. Conversely, other studies have failed to detect significant differences or have observed increased HO-1 immunoreactivity in placental specimens, potentially reflecting a compensatory response to oxidative stress [138]. This apparent contradiction underscores the dynamic and context-dependent regulation of HO-1 during pregnancy complications and highlights the clinical heterogeneity of PE.

Beyond CO, bilirubin, another HO-1-derived metabolite, appears to be relevant to PE pathophysiology. Lower bilirubin concentrations have been associated with adverse maternal and neonatal outcomes, implying that diminished antioxidant buffering may create a pro-oxidant intrauterine environment. Iron metabolism dysregulation is also frequently observed in PE [139]. Meta-analyses have consistently shown elevated serum iron levels in affected individuals, and excess free iron can exacerbate oxidative injury [140,141]. Moreover, early iron supplementation has been linked to an increased risk of gestational hypertension, further emphasizing the need for careful management of iron homeostasis in pregnancy.

At a mechanistic level, HO-1 has been shown to exert negative regulatory effects on key anti-angiogenic factors implicated in PE development. Experimental studies demonstrate that HO-1 can suppress the release of soluble fms-like tyrosine kinase-1 (sFlt-1) and soluble endoglin (sEng), both of which antagonize vascular endothelial and placental growth factors [142]. Inhibition of HO-1 in animal models leads to hypertension, oxidative damage, and impaired placental perfusion, whereas pharmacological induction of HO-1 reverses these alterations [143]. Clinically, statins such as pravastatin have been shown to upregulate HO-1 expression in endothelial cells and reduce sFlt-1 production, supporting their potential role as therapeutic agents to restore angiogenic balance in PE [142].

Taken together, these findings highlight HO-1 as a central integrator of iron–immune crosstalk and redox regulation in preeclampsia. Whether diminished HO-1 activity represents a primary defect or a maladaptive response remains to be determined. Further studies are warranted to elucidate how HO-1-derived metabolites, iron homeostasis, and inflammatory pathways collectively contribute to disease severity and clinical outcomes.

Pro-inflammatory cytokines that respond to iron status—IL-6 and TNF-α in particular—are markedly elevated in PE and correlate with disease severity. IL-6 is a key inducer of the liver hormone hepcidin (which regulates iron sequestration), linking inflammation to iron metabolism [144,145]. PE patients, especially those with severe features, exhibit higher circulating IL-6 levels compared to those with mild PE or normotensive pregnancies, with concentrations rising in parallel with clinical severity (e.g., higher in early-onset PE or cases with significant hypertension). Likewise, TNF-α, a classic M1 macrophage cytokine, is elevated in PE and contributes to endothelial dysfunction; higher TNF-α levels are associated with more pronounced maternal symptoms and fetal growth restriction [146,147]. These cytokines not only serve as biomarkers of severity but also actively worsen the condition by promoting leukocyte activation, endothelial cell injury, and coagulation abnormalities. Notably, IL-6 and TNF-α can further disturb iron homeostasis. Hepcidin is a hepatic peptide hormone that serves as the central regulator of systemic iron metabolism and plays a pivotal role in coordinating iron homeostasis during pregnancy. Under physiological conditions, maternal hepcidin levels decline markedly compared to the non-pregnant state, facilitating increased dietary iron absorption and efficient placental transfer to meet fetal demands [148]. However, inflammatory disorders such as preeclampsia (PE) can profoundly disrupt this balance. Experimental models have shown that hepcidin negatively regulates iron uptake in the intestine and iron release from macrophages, as evidenced by mouse studies demonstrating that hepcidin deficiency leads to uncontrolled intestinal absorption and iron overload, while overexpression results in severe systemic and placental iron restriction [149]. Specifically, transgenic mice engineered to overproduce hepcidin in the liver die shortly after birth due to profound iron deficiency, highlighting that hepcidin can restrict iron transport not only across the gut epithelium but also through the placenta [149].

Clinical observations in PE have revealed substantial variability in hepcidin dynamics, reflecting the complex interplay between inflammation, iron status, and placental function. Several studies have documented elevated maternal serum hepcidin levels in PE compared to normotensive pregnancies. This increase is often attributed to inflammation-driven upregulation, primarily mediated by rapid interleukin-6 (IL-6)-induced hepatic synthesis [146,150]. In this setting, excessive hepcidin can trigger ferroportin degradation, blocking iron export from macrophages and enterocytes, causing iron sequestration, limited fetal iron availability, and exacerbated placental oxidative stress [149,151]. A meta-analysis pooling multiple studies confirmed that serum hepcidin is, on average, significantly higher among women who develop PE, supporting the connection between inflammatory activation and iron-restrictive responses [152].

Interestingly, some PE cases present both elevated hepcidin and elevated serum iron, a seemingly paradoxical pattern possibly arising from simultaneous hemolysis-related iron release and inflammatory hepcidin induction [153]. Such elevated hepcidin may represent an attempted defense to limit iron-mediated oxidative damage, though this response frequently fails to prevent endothelial dysfunction. Conversely, other studies have found suppressed hepcidin in subsets of PE patients despite marked inflammation and iron loading [154]. For example, severe or early-onset PE has been associated with low hepcidin levels, potentially reflecting the dominant inhibitory effects of placental hypoxia overriding inflammatory signals [154]. In such scenarios, reduced hepcidin can permit excessive iron efflux into circulation and the placenta, promoting iron overload and oxidative injury. Discrepancies among studies likely stem from heterogeneity in study populations, gestational age at sampling, disease severity, and iron supplementation practices.

Overall, the regulation of iron-responsive immune pathways in PE appears highly context-dependent and is shaped by a complex interplay among inflammatory mediators such as IL-6 and TNF-α, dysregulated hepcidin signaling, aberrant iron handling, and impaired antioxidant defenses mediated by HO-1 deficiency. These interconnected mechanisms contribute to oxidative stress, endothelial dysfunction, and placental maladaptation, collectively driving disease severity and heterogeneity. The apparent contradictions among studies—particularly regarding HO-1 expression patterns and hepcidin levels—highlight the dynamic and multifactorial regulation of iron metabolism during pregnancy complications. Future investigations stratified by PE subtype, gestational age, inflammatory status, and iron exposure will be essential to clarify how these iron–immune interactions jointly influence maternal–fetal iron distribution, oxidative injury, and clinical outcomes, and to identify targeted strategies for therapeutic modulation.

### 6.2. Targeting Oxidative Stress and Angiogenic Imbalance via the Nrf2/HO-1 Pathway

One major approach involves pharmacologic induction of heme oxygenase-1 (HO-1) and reinforcement of its upstream regulatory pathways. Statins, in particular, have emerged as promising candidates due to their capacity to modulate HO-1 expression [142]. Simvastatin has been shown to upregulate HO-1 mRNA and protein in endothelial cells, leading to the suppression of sFlt-1 and sEng, two anti-angiogenic mediators central to PE pathophysiology. This regulatory effect appears to be specific to the cholesterol biosynthesis pathway and is reversible upon supplementation with farnesyl pyrophosphate. While high-dose statins are contraindicated during pregnancy due to teratogenic concerns observed in animal studies, retrospective data from familial hypercholesterolemia cohorts have not demonstrated a significant increase in congenital anomalies when statin therapy was inadvertently continued. These findings support the rationale for further investigation of statins as disease-modifying agents to restore angiogenic balance via HO-1 induction.

Pharmacologic induction of HO-1 has also been explored using cobalt protoporphyrin, an established HO-1 agonist [155]. In a rat model of placental ischemia induced by reduced uterine perfusion pressure, cobalt protoporphyrin treatment attenuated hypertension, decreased placental oxidative stress, and restored pro-angiogenic signaling, as evidenced by normalization of sFlt-1/VEGF ratios and reduced superoxide production [155]. These data further support the therapeutic potential of HO-1 activation to counteract placental ischemia and endothelial dysfunction.

Additionally, modulators of upstream regulatory pathways such as CD151 have garnered attention. CD151 expression is diminished in preeclamptic placentas and appears to govern antioxidant gene transcription, including HO-1. Experimental models demonstrated that restoring CD151 expression in trophoblasts increases HO-1 and other antioxidant defenses, whereas CD151 downregulation induces oxidative stress, apoptosis, and phenotypic features reminiscent of PE [137]. This positions CD151 as a prospective target to enhance endogenous antioxidant capacity.

Natural compounds capable of engaging the Nrf2/HO-1 axis are also under investigation. Liensinine, an isoquinoline alkaloid, has demonstrated protective effects in experimental gestational hypertension by activating Nrf2 and HO-1, resulting in reduced oxidative injury and inflammation as well as improved placental histopathology [156]. Similarly, astaxanthin supplementation in PE models mitigated oxidative damage and inflammatory signaling, downregulated pro-apoptotic markers, and restored HO-1 expression [157].

Collectively, these studies provide compelling evidence that therapeutic strategies aimed at inducing HO-1 or reinforcing its upstream regulators may alleviate the oxidative and inflammatory milieu characteristic of preeclampsia, offering a promising avenue for disease prevention and treatment, as summarized in Table 1.

### 6.3. Inhibition of Ferroptosis to Preserve Trophoblast Function

Ferroptosis has emerged as a central mechanism contributing to trophoblast dysfunction and vascular injury in PE. Diverse therapeutic strategies have been explored to inhibit ferroptosis and alleviate placental oxidative stress. Small-molecule ferroptosis inhibitors such as Ferrostatin-1 have been shown to reverse key pathological features, including impaired spiral artery remodeling and excessive lipid peroxidation, in animal models with FTL deficiency or hypoxia-induced placental injury [158,159]. Additional studies in trophoblast cultures treated with erastin demonstrated that Ferrostatin-1 improved cell viability and reduced reactive oxygen species accumulation [160,161].

Natural compounds have also attracted attention as potentially safer options during pregnancy. Quercetin, for example, ameliorated endothelial dysfunction and inflammation in PE models by targeting epidermal growth factor receptor (EGFR) signaling [162]. Extracts of Amomum villosum and their main component, vanillic acid, were shown to enrich beneficial gut microbes such as Bifidobacterium bifidum, restore placental antioxidant proteins including GPX4, FTH1, and cystine/glutamate antiporter (xCT), and improve maternal–fetal outcomes in Nω-nitro-L-arginine methyl ester (L-NAME)-induced PE mice [163].

Modulation of antioxidant pathways has proven particularly promising. Rosiglitazone, a PPARγ agonist, activated Nrf2-dependent transcription of GPX4, alleviating lipid oxidation and ferroptosis in hypoxia-exposed trophoblasts [164]. Similarly, upregulation of Parkinson disease protein 7 (DJ-1) enhanced Nrf2 stability and counteracted ferroptosis in placental tissues and BeWo cells [165]. Inhibition of Nox2 restored GPX4 and STAT3 expression while reducing mitochondrial dysfunction [166]. Elabela, a placental peptide, prevented ferritinophagy and preserved FTH1-mediated iron storage, thereby suppressing ferroptosis in both mouse models and trophoblast cultures [167].

Conversely, several pro-ferroptotic factors have been identified. Upregulation of p53 was observed to downregulate SLC7A11 and GPX4, exacerbating iron-dependent lipid peroxidation in trophoblasts [168]. Mixed-lineage leukemia protein 1 (MLL1) promoted ferroptosis by increasing RNA binding motif protein 15 (RBM15) expression, leading to destabilization of tripartite motif containing 72 (TRIM72) and accumulation of a disintegrin and metalloproteinase domain 9 (ADAM9) [169]. Methyltransferase-like 3 (METTL3)-mediated N6-methyladenosine (m6A) methylation enhanced ACSL4 mRNA stability and drove ferroptotic cell death under hypoxic stress [51].

Meanwhile, reduced Wilms tumor 1-associated protein (WTAP) expression impaired SRY-box transcription factor 2 (SOX2)-dependent transcription of antioxidant genes, further sensitizing trophoblasts to ferroptosis [170]. Butyrophilin subfamily 3 member A2 (BTN3A2) increased ferroptosis by repressing milk fat globule-EGF factor 8 protein (MFGE8) in endothelial cells [171], and elevated TLR4 and pannexin 1 (Panx1) expression in placental tissues correlated with decreased SLC7A11 and GPX4 levels [172]. Additionally, exposure to 25-hydroxycholesterol triggered lipid peroxidation, apoptosis, and ferroptosis in trophoblast cell lines [173].

Interventions targeting noncoding RNAs and post-transcriptional regulation have also yielded compelling results. Knockdown of T-cell leukemia/lymphoma 6 (TCL6) reduced ferroptosis by limiting transferrin receptor (TFRC)-mediated iron accumulation in LPS-treated trophoblasts [174]. Silencing miR-30b-5p alleviated hypoxia-induced ferroptosis by restoring SLC7A11 and ferroportin expression [160], whereas inhibition of miR-106b-5p upregulated GPX4 and FTH1 via the ACSL4 axis [161].

Finally, immune-related pathways are implicated in ferroptosis modulation. Caspase-6 activation in macrophages promoted HMGB1 secretion and triggered ferroptosis in adjacent trophoblasts, while Caspase-6 knockdown ameliorated placental injury in reduced uterine perfusion pressure (RUPP)-induced PE models [175]. Taken together, these studies illustrate that targeting ferroptosis—via pharmacological inhibitors, natural products, gene regulation, and modulation of immune and metabolic pathways—holds significant promise for developing new therapeutic options to improve pregnancy outcomes in PE, as summarized in Table 2.

### 6.4. Modulating Macrophage Polarization and Iron–Immune Crosstalk

Recent advances in understanding the molecular interplay between iron metabolism, ferroptosis, and macrophage polarization have highlighted promising avenues to alleviate placental dysfunction and systemic inflammation in preeclampsia (PE). Although studies directly addressing PE remain limited, extensive research in inflammatory, metabolic, and vascular disease models provides compelling mechanistic insights that can be extrapolated to inform potential interventions.

Dysregulated iron homeostasis is increasingly recognized as a contributor to oxidative stress and trophoblast injury in PE. In murine models of atherosclerosis induced by high-fat diets, inhibition of hepcidin markedly reduced macrophage iron accumulation and inflammatory activity, suggesting that targeting systemic iron regulation could mitigate pathological iron loading in the placenta [149]. In parallel, studies utilizing cecal ligation and puncture (CLP)-induced sepsis and hepatic injury models demonstrated that suppression of NCOA4-dependent ferritinophagy attenuates ferroptosis and STING-mediated inflammatory signaling in macrophages and monocytes [177]. In the specific context of PE, recent investigations employing the reduced uterine perfusion pressure (RUPP) rat model and macrophage–trophoblast co-cultures showed that silencing Caspase-6 in macrophages limited ferroptosis in trophoblasts and improved maternal disease phenotypes [175].

Iron chelation and antioxidant strategies have likewise gained traction as candidate therapeutic approaches. For example, uridine supplementation activated Nrf2 signaling and suppressed lipid peroxidation, thereby reducing macrophage ferroptosis in LPS-induced acute lung injury models and THP-1-derived macrophages [178]. GCH1 overexpression enhanced ferroptosis resistance in RAW264.7 macrophages stimulated with LPS, partly through AMPK pathway activation [179]. Moreover, the development of multifunctional nanomaterials such as ceria nanozymes coordinated with curcumin has shown dual capability to scavenge reactive oxygen species and promote anti-inflammatory macrophage polarization in LPS-induced sepsis-associated cardiac injury [180].While these interventions have primarily been evaluated in systemic inflammatory disorders, their underlying mechanisms are relevant to placental oxidative stress and immunopathology. Aberrant macrophage polarization contributes significantly to the persistence of a pro-inflammatory microenvironment in PE. Single-cell RNA sequencing and in vivo galectin-9 administration in pregnant mouse models have revealed that trophoblast-derived galectin-9 drives the accumulation of pro-inflammatory CD11c^high^ decidual macrophages, impairing spiral artery remodeling [181]. Consequently, blocking galectin-9/CD44 interactions represents an attractive immunomodulatory target.

Strategies to induce M2-like anti-inflammatory macrophage phenotypes have demonstrated efficacy across multiple disease models. In vitro studies of human monocyte-derived macrophages indicated that nitric oxide-donating pravastatin derivatives effectively suppress NF-κB activation and pro-inflammatory cytokine release [182], whereas 15d-PGJ2 promoted M2 polarization markers in bone marrow-derived macrophages [183]. In high-fat diet-fed ApoE^−/−^ mice, quercetin supplementation alleviated brain iron deposition and corrected microglial M1/M2 imbalance, reducing ferroptosis-associated neuroinflammation [184]. Furthermore, nanoparticle-based delivery of TLR agonists has successfully reprogrammed tumor-associated macrophages in murine cancer models, providing proof-of-concept for precision targeting of macrophage phenotypes [185].

Ferroptosis not only inflicts direct cellular injury but also propagates inflammation through the release of damage-associated molecular patterns, notably HMGB1. In collagen-induced arthritis and collagen antibody-induced arthritis mouse models, ferroptotic M2 macrophages released HMGB1, which engaged TLR4 and activated STAT3 signaling in M1 macrophages, fueling inflammatory amplification loops [186]. Similar HMGB1–TLR4–STAT3 interactions have been observed in CLP-induced septic lung injury models and LPS-stimulated BEAS-2B–THP-1 co-cultures [187].Ferroptosis inhibitors such as liproxstatin-1 not only preserved M2 macrophage populations but also attenuated tissue injury in erythroid Jak2V617F-driven iron-overload atherosclerosis and serum-transfer arthritis models [188,189]. Lipid peroxidation has emerged as a critical node in ferroptosis execution. In bleomycin-induced systemic sclerosis, pharmacologic inhibition of ACSL4 effectively reduced ferroptosis and fibrosis in vivo and in activated macrophages [190]. Similarly, Nrf2 activation was shown to decrease ferroptosis susceptibility in BV2 microglial cells and LPS-challenged mice [191].

Together, these findings highlight that interventions stabilizing antioxidant defenses and modulating ferroptosis may offer therapeutic benefits in PE, where trophoblast injury and macrophage dysfunction converge. Research in metabolic dysfunction-associated steatohepatitis has shown that macrophage-derived oxidized phospholipids stimulate hepatocyte hepcidin production, which in turn drives Kupffer cell iron loading and ferroptosis, underlining the importance of iron–immune crosstalk in chronic inflammation [131]. In neurodegenerative disease contexts, iron accumulation and inflammasome activation were found to promote microglial M1 polarization and sensitize M2 phenotypes to ferroptosis [192]. Additionally, studies of erythroid lineage Jak2V617F mutant mice demonstrated that erythrophagocytosis by plaque macrophages precipitates ferroptosis and exacerbates vascular damage [188]. Although these models were not developed in the context of pregnancy, they reinforce the conserved nature of iron-dependent immunopathology and support their translational relevance to PE.

Collectively, these studies provide a robust rationale for integrated therapeutic strategies combining iron chelation, ferroptosis inhibition, and immunometabolic reprogramming of macrophages, as summarized in Table 3. While additional research is needed to validate these approaches in PE-specific experimental systems, the convergence of mechanistic evidence across cardiovascular, rheumatologic, hepatic, and neuroinflammatory disease models underscores the translational promise of targeting the iron–immune axis as an innovative paradigm to improve maternal and fetal outcomes. Nevertheless, it should be acknowledged that most findings derive from non-pregnant models or in vitro studies, and their relevance to human pregnancy requires further confirmation through dedicated preclinical and clinical investigations.

## 7. Conclusions and Future Perspectives

Preeclampsia remains a leading cause of maternal and perinatal morbidity worldwide, with its complex and multifactorial pathogenesis only partially understood. Traditional models centered on impaired trophoblast invasion and angiogenic imbalance have been substantially enriched by recent insights into the interplay between iron metabolism and immune regulation at the maternal–fetal interface. Accumulating evidence demonstrates that iron overload, oxidative stress, and ferroptosis disrupt trophoblast function and vascular remodeling, while iron-responsive immune pathways, particularly those involving macrophage polarization and inflammatory cytokine release, exacerbate placental dysfunction and systemic endothelial injury. Macrophages, as both sensors and regulators of the iron microenvironment, are emerging as central players in this process. The pathological polarization toward pro-inflammatory M1-like phenotypes, combined with impaired efferocytosis and iron sequestration, creates a self-perpetuating cycle of inflammation, oxidative injury, and immunometabolic dysregulation. This iron–immune axis not only correlates with disease severity but also offers promising biomarkers and therapeutic targets for early diagnosis and intervention.

First, advancing our understanding of the molecular drivers of ferroptosis in preeclamptic trophoblasts could reveal specific therapeutic targets. Identifying key regulatory factors, such as GPX4, SLC7A11, and other antioxidant systems that protect cells from ferroptosis, could enable interventions to mitigate oxidative stress within the placenta. Additionally, research should focus on the spatial and temporal expression of ferroptosis-related markers throughout pregnancy to determine how their dysregulation contributes to the onset and progression of preeclampsia. Another promising research direction involves further characterizing the relationship between macrophage phenotypes and ferroptotic activity in the placenta. The traditional M1/M2 polarization model is now considered overly simplistic, as placental macrophages exhibit a spectrum of activation states, reflecting dynamic changes in the local microenvironment. Future studies using single-cell RNA sequencing and proteomics could provide more granular insights into how ferroptosis shapes macrophage behavior across pregnancy stages and in response to PE-associated stressors. A comprehensive understanding of macrophage subpopulations and their functional roles could enable researchers to identify specific subsets amenable to therapeutic modulation for restoring immune balance in the placenta. Furthermore, the role of extracellular vesicles (EVs) and exosomes in mediating ferroptosis-driven macrophage signaling requires systematic investigation. EVs carrying lipid peroxides, iron, and other markers of oxidative stress may facilitate crosstalk between ferroptotic trophoblasts and placental macrophages, effectively propagating inflammatory signals within the placental environment. Targeting the release or uptake of these vesicles may offer a strategy for disrupting these harmful signaling pathways, thereby reducing placental inflammation and improving maternal–fetal health outcomes. Finally, translational research is essential to bridge the gap between foundational studies and clinical applications. Developing non-invasive biomarkers that reflect ferroptosis and macrophage activity could enable early diagnosis and monitoring of preeclampsia. For instance, detecting specific ferroptosis-related metabolites, lipid peroxidation products, or macrophage-derived cytokines in maternal blood could provide valuable diagnostic insights. Intervention studies focusing on iron chelation therapies, antioxidant treatments, or immunomodulators are also promising.

By intervening at the level of iron metabolism, oxidative stress management, or immune modulation, it may be possible to prevent or alleviate the adverse health impacts of preeclampsia on both mother and fetus. In conclusion, the interactions between ferroptosis and macrophages in normal pregnancy and preeclampsia provide a rich field of research with significant clinical implications. By unraveling the complex networks regulating cell death and immune responses within the placenta, future studies can lay the groundwork for targeted therapies aimed at improving pregnancy outcomes and maternal–fetal health.

## Figures and Tables

**Table 1:** Summary of HO-1-related therapeutic strategies and mechanisms in preeclampsia models.

	Mechanisms	Samples	Ref.
Astaxanthin	Suppresses oxidative stress and inflammation via inhibition of ROS/NF-κB/ROCK II and activation of HO-1	H_2_O_2_-treated HUVECs; L-NAME-induced preeclamptic rats	[157]
CD151	Regulates antioxidant gene expression via ERK/Nrf2 pathway; its downregulation increases oxidative stress and apoptosis	Human preeclamptic placentas; CD151-knockdown trophoblast cells; CD151-deficient mouse model	[137]
Cobalt protoporphyrin	Reduces sFlt-1, endothelin-1, and superoxide via HO-1/CO/bilirubin pathway; restores angiogenic balance and lowers blood pressure	RUPP-induced placental ischemia rat model; normal pregnant rats	[155]
Liensinine	Activates Nrf2/HO-1 pathway to reduce oxidative stress, inflammation, and placental damage; promotes VEGF and PIGF expression	L-NAME-induced gestational hypertension model in Wistar rats	[156]
Statins	Upregulates HO-1, which inhibits sFlt-1 and sEng release via CO-mediated suppression of VEGFR-2 signaling	Endothelial cells; placental villous explants; HO-1 knockout mice	[142]

**Table 2:** Summary of ferroptosis-related therapeutic targets and mechanisms in preeclampsia.

	Mechanisms	Samples	Ref.
25-hydroxycholesterol	Induces oxidative stress, leading to ferroptosis, apoptosis, and autophagy via mitochondrial ROS, lipid peroxidation, GSH depletion, and MMP loss	Swan 71 EVT cell line treated with 25HC; rescue by Z-VAD-FMK and ferrostatin-1	[173]
Amomum villosum Lour	Enhances gut *B. bifidum* via vanillic acid → restores gut–placenta barrier and inhibits placental ferroptosis by upregulating FPN1, FTH1, xCT, and GPX4	L-NAME-induced PE mouse model; FMT; in vitro and in vivo validation with vanillic acid and *B. bifidum*	[163]
ANXA1	Suppresses trophoblast ferroptosis by downregulating KISS1	RSL3-induced ferroptosis in trophoblasts; PE-like mouse model treated with Ac2–26	[176]
BTN3A2	Promotes ferroptosis and inhibits angiogenesis via interaction with MFGE8 and suppression of MFGE8 expression	PE patient placentas; hypoxia-treated HUVECs; L-NAME-induced PE rat model	[171]
DJ-1	Activates Nrf2/GPX4 signaling to reduce lipid peroxidation and inhibit ferroptosis in trophoblasts	Human placental tissues (PE vs. control); BeWo cells; RSL3/Fer-1 treatments; DJ-1^+/+^ and DJ-1^−/−^ cell models	[165]
Elabela	Inhibits ferroptosis by blocking ferritinophagy, increasing FTH1, reducing labile iron pool and lipid peroxidation	Human PE placentas; PE-like mouse model; Erastin-treated trophoblasts (in vitro)	[167]
Ferrostatin-1	Inhibits FTL deficiency-induced ferroptosis by reducing lipid peroxidation and restoring spiral artery remodeling and blood pressure	FTL-knockdown pregnant rat model; ferrostatin-1 treatment group	[159]
HOTAIR (lncRNA)	Knockdown of HOTAIR upregulates miR-106b-5p, downregulates ACSL4, restores mitochondrial membrane potential, and inhibits H_2_O_2_-induced ferroptosis	HTR-8/SVneo trophoblast cell line; H_2_O_2_-induced oxidative injury model	[161]
Macrophages Caspase-6	Knockdown of Caspase-6 reduces HMGB1 signaling, inhibits macrophage-induced ferroptosis in trophoblasts	PE rat model (RUPP); co-culture of THP-1 macrophages and HTR-8/SVneo trophoblasts; si-Caspase-6 and anti-HMGB1 treatments	[175]
METTL3	Promotes ACSL4 mRNA stability via m^6^A modification, increases ferroptosis, and impairs trophoblast migration and invasion	Human PE placental tissues; hypoxia-stimulated trophoblasts; PE rat model with METTL3 knockdown	[51]
miR-30-5p	Induces ferroptosis by downregulating xCT (Cys2/Glu antiporter), PAX3, and FPN1, leading to GSH depletion and Fe^2+^ accumulation	Human PE placental tissues; hypoxia-treated trophoblasts; PE rat model with miR-30b-5p inhibition and ferroptosis inhibitors	[160]
MLL1	Promotes ferroptosis by epigenetically upregulating RBM15 (via H3K4me3), which represses TRIM72, leading to ADAM9 degradation and trophoblast ferroptosis	PE mouse model; Erastin-induced ferroptosis in HTR-8/SVneo cells; ChIP, RIP, and Co-IP assays	[169]
NOC2L	Inhibits ferroptosis by suppressing p53 activity, upregulating SLC7A11 and GPX4, reducing Fe^2+^, MDA, and lipid ROS	Hypoxia-stimulated HTR-8/SVneo cells; PE rat model; p53 inhibitor (PFT-α) and ferrostatin-1 intervention	[158]
Nox2	Promotes ferroptosis by inhibiting STAT3 and GPX4; knockdown reduces ROS, lipid peroxidation, mitochondrial dysfunction, and restores angiogenesis and glycolysis balance	PE placental tissues; trophoblast cells (in vitro); Seahorse ECAR/OCR assays	[166]
p53	Inhibits SLC7A11 and GPX4 expression, promotes ferroptosis and oxidative stress, and impairs placental angiogenesis (VEGFA, PLGF↓; VEGFR1↑)	PE placental tissues; trophoblasts (p53^+/+^ vs. p53^−/−^); Erastin and Fer-1 treatment; PE rat model	[168]
PPARγ (agonist: Rosiglitazone)	Activates PPARγ/Nrf2 signaling to reduce lipid peroxidation and trophoblast ferroptosis; regulates lipid oxidation (not SREBP1-driven lipid synthesis)	PE patient serum and placentas; hypoxia/erastin-treated trophoblasts; PE rat model; si-Nrf2 and Rosiglitazone intervention	[164]
Quercetin	Binds to EGFR to inhibit ferroptosis and inflammation, restores endothelial function, and improves PE symptoms	sRUPP-induced PE rat model; in vitro endothelial dysfunction models; EGFR targeting validation	[162]
TCL6 (lncRNA)	Knockdown of TCL6 reduces ferroptosis and inflammation by sponging miR-485-5p, thereby downregulating TFRC; improves viability, migration, and invasion of trophoblasts	HTR-8/SVneo cells; LPS-induced injury model; TCL6, miR-485-5p, and TFRC expression manipulation	[174]
TLR4 & Panx1	Promote ferroptosis by suppressing SLC7A11 and reducing GSH, GPX4, and HO-1 levels; positively correlate with Fe^2+^, MDA, ATF3; potential diagnostic markers in PE	Human PE placental tissues (*n* = 65) and healthy controls (*n* = 25); serum ELISA for Panx1 and TLR4; RT-PCR for placental ferroptosis markers	[172]
WTAP	Enhances SOX2 mRNA stability via m^6^A modification, leading to upregulation of GPX4, SLC7A11, and FTH1; inhibits ferroptosis and alleviates PE symptoms	PE patient placentas (*n* = 20); hypoxia-treated HTR-8/SVneo cells; PE rat model	[170]

**Table 3:** Therapeutic Strategies Targeting Macrophage Ferroptosis Across Inflammatory Disease Models.

	Mechanisms	Samples	Ref.
Anti-TNF/IL-6/Hepcidin	Anti-TNF therapy reduces IL-6 and prohepcidin levels, improving iron availability by relieving macrophage iron retention in ACD	Sera from 21 IBD patients (inflammatory bowel disease with anemia of chronic disease)	[193]
ACSL4 (inhibitor)	Inhibition of ACSL4 reduces inflammatory macrophage ferroptosis via the calpain/ACSL4 axis, alleviating fibrosis in SSc; LPS increases ferroptosis sensitivity in activated macrophages	BLM-induced systemic sclerosis mice; BMDMs and Raw264.7 macrophages (±calpain modulation; ±LPS)	[190]
Caspase-6	Caspase-6 knockdown in macrophages reduces trophoblast ferroptosis by inhibiting HMGB1-mediated macrophage–trophoblast signaling	RUPP-induced PE rat model; THP-1 and HTR8/SVneo co-culture system	[175]
Ceria nanozyme + Curcumin (CeCH)	Scavenges ROS (SOD/CAT-like), inhibits RSL3-induced ferroptosis in cardiomyocytes, promotes M2 macrophage polarization, reduces LPS-induced inflammation and cardiac injury	RSL3-induced cardiomyocyte ferroptosis model; LPS-induced sepsis mouse model; in vitro M1/M2 macrophage polarization assay	[180]
Galectin-9/CD44 axis	Trophoblast-derived Galectin-9 binds to CD44, activating CD11c^++^ decidual macrophages, which impairs spiral artery remodeling and contributes to the pathogenesis of preeclampsia.	Human placentas, recombinant Gal-9-induced PE mouse model	[181]
GCH1	GCH1 inhibits ferroptosis and reduces M1 polarization and inflammatory cytokine release in LPS-stimulated alveolar macrophages, partly via AMPK signaling pathway suppression	LPS-stimulated RAW264.7 macrophages; GCH1-knockdown; GSE40885/GSE112720 datasets	[179]
Hepcidin deficiency	Hepcidin deficiency reduces macrophage iron levels, suppresses M1 polarization and inflammation, and protects against atherosclerosis	THP1 Cells, Hamp^−/−^/Ldlr^−/−^ mice	[149]
HMGB1/TLR4/STAT3 axis	Ferroptotic M2 macrophages release HMGB1, which activates TLR4/STAT3 signaling in M1 macrophages, driving synovial inflammation	CIA and CAIA mouse models of rheumatoid arthritis	[186]
Irf7	Irf7 transcriptionally activates Srg3, which promotes NF-κB signaling, M1 macrophage polarization, and ferroptosis, exacerbating ALI	CLP-induced rat model of sepsis-induced ALI; LPS-treated BEAS-2B + THP-1 co-culture system	[187]
Liproxstatin-1	Inhibits macrophage ferroptosis induced by phagocytosis of oxidized RBCs; reverses lipid peroxidation, endothelial damage, and plaque necrosis	VFEpoR mice (erythroid-specific Jak2V617F), Jak2VF chimeric mice	[188]
NCF1	NCF1 in macrophages increases oxidized phospholipids, activates TLR4-dependent hepcidin release from hepatocytes, causing iron overload and ferroptosis in Kupffer cells	Human MASH samples; MASH mouse model	[131]
NCX 6550 (NO-donating pravastatin)	Inhibits NF-κB translocation and cytokine (TNF-α, IL-6) release; enhances PPARγ expression in monocytes/macrophages	Human primary monocytes and monocyte-derived macrophages	[182]
Nrf2	RSL3 upregulates Nrf2, inhibits Pol II recruitment to cytokine gene promoters, reduces inflammation and enhances ferroptosis resistance	Microglia (BV2), peritoneal macrophages, LPS-induced inflammation model in mice	[191]
PPAR-γ	PPAR-γ activation induces M2 macrophage polarization and suppresses cytokine release and neutrophil migration via HO-1 upregulation	Bone marrow-derived macrophages (BMDM), carrageenan-induced inflammation model	[183]
Quercetin	Quercetin reduces iron accumulation and inhibits neuronal ferroptosis and pyroptosis by shifting microglia from pro-inflammatory M1 to anti-inflammatory M2 phenotype	ApoE^−^/^−^ mice fed high-fat diet; ox-LDL + iron-treated microglia-neuron co-culture	[184]
STING–NCOA4/HET0016	STING binds NCOA4 to promote ferritinophagy, enhancing macrophage ferroptosis and inflammatory responses in sepsis; HET0016 blocks this interaction and reduces mortality	Septic mouse model; PBMCs from septic patients	[177]
TAK1	Hepatocyte-specific TAK1 deletion induces ferroptosis and oxidative DNA damage; this activates macrophage cGAS-STING signalling, leading to inflammation, fibrosis, and tumorigenesis; STING inhibitor and Fer-1 reduce damage	Hepatocyte-specific TAK1 knockout mice (TAK1ΔHEP); high-iron diet model	[194]
TLR agonist-loaded nanoparticles (PNP@R@M-T)	Selective delivery of TLR agonists reprograms M2-like TAMs into M1 phenotype, reducing tumor-supportive immunosuppression and enhancing antitumor immunity	In vivo tumor model; TAMs in tumor microenvironment	[185]
Uridine/UPP1/Nrf2	Uridine activates Nrf2 signaling, upregulates SLC7A11, GPX4, HO-1, and suppresses ACSL4 expression to inhibit macrophage ferroptosis, reducing inflammation and oxidative injury	LPS-induced ALI mouse model and THP-1 macrophages	[178]

## Abbreviations

Abbreviations listed in alphabetical order

4-HNE	4-hydroxynonenal
ACOG	American College of Obstetrics and Gynecology
ACSL4	acyl-CoA synthetase long-chain family member 4
BH4	tetrahydrobiopterin
CCL	C-C motif chemokine ligand
CCR2	C-C chemokine receptor type 2
CD151	cluster of differentiation 151
CO	carbon monoxide
CoQ10	coenzyme Q10
CXCL	C-X-C motif chemokine ligand
DJ-1	Parkinson disease protein 7
DMs	Decidual macrophages
DMT1, SLC11A2	divalent metal transporter 1
dNK	natural killer
EP	erythrophagocytosis
FPN, SLC40A1	ferroportin
FSP1	ferroptosis suppressor protein 1
FTH1	ferritin heavy chain 1
FTL	Ferritin light chain 1
FTN	ferritin
GDM	gestational diabetes mellitus
GM-CSF	granulocyte-macrophage colony-stimulating factor
GPXs	glutathione peroxidases
GSH	glutathione
HA	hemophiliac arthritis
HAMP	hepcidin antimicrobial peptide
HMGB1	high-mobility group box 1
HO-1	heme oxygenase-1
holo-Tf	Fe3+-loaed Transferrin-bound
HSCs	hematopoietic stem cells
IFN-γ	interferon-gamma
IL-6	interleukin-6
iNOS	inducible nitric oxide synthase
IRF	interferon regulatory factor
IRP-IRE	iron regulatory protein–iron-responsive element
IUGR	intrauterine growth restriction
JMJD3	jumonji domain containing 3
LPS	lipopolysaccharide
MAPK	mitogen-activated protein kinase
MDA	malondialdehyde
MMPs	Matrix metalloproteases
NADPH	nicotinamide adenine dinucleotide phosphate
NCF1	neutrophil cytosolic factor 1
NCOA4	nuclear receptor coactivator 4
NF-κB	nuclear factor kappa B
NK cells	natural killer (NK) cells
NOS	nitric oxide synthases
NOX2	NADPH oxidase 2
NRF2	nuclear factor erythroid 2–related factor 2
NTBI	non-transferrin-bound iron
O2−	superoxide anion
P53	tumor protein p53
PCBP2	poly-(rC)-binding protein 2
PE	preeclampsia
PGE2	prostaglandin E2
PLOOHs	phospholipid hydroperoxides
PPAR	peroxisome proliferator-activated receptor
ROS	Reactive oxygen species
sEng	soluble endoglin
sFlt-1	soluble fms-like tyrosine kinase-1
Sry	sex-determining region Y
STAT	signal transducer and activator of transcription
STBs	syncytiotrophoblastics
STEAP3	six-transmembrane epithelial antigen of the prostates 3
TfR1	transferrin receptor 1
TGF-β	transforming growth factor-beta
THP-1	human monocytic leukemia cell line
TLR	Toll-like receptor
TNF-α	tumor necrosis factor-alpha
UTRs	Untranslated regions
VEGF	vascular endothelial growth factor
ZIP14, SLC39A14	Zrt- and Irt-like protein 14
ZIP8, SLC39A8	Zrt- and Irt-like protein 8
